# Is Medicaid Payment the Key to Raising Staffing Levels in Nursing Homes? Insights from a Facility Level Analysis

**DOI:** 10.1093/geroni/igaf122.2691

**Published:** 2025-12-31

**Authors:** Edward Miller, John Bowblis, Elizabeth Simpson, Marc Cohen

**Affiliations:** University of Massachusetts Boston, Boston, Massachusetts, United States; Miami University, Oxford, Ohio, United States; University of Massachusetts Boston, Boston, Massachusetts, United States; University of Massachusetts Boston, Boston, Massachusetts, United States

## Abstract

Nursing staff levels are associated with nursing home (NH) quality, but NHs face substantial challenges recruiting and retaining nursing staff. Medicaid, which does not cover the cost of care, is the primary payer for approximately two-thirds of residents, constraining NHs from increasing staff. While past work has studied state-level Medicaid payment rates, there is little work examining the relationship between staffing levels and facility-level payments. This study utilized data from the Payroll-Based Journal data along with Medicaid payment rates collected from states to examine the association between Medicaid rates and staffing levels, and to determine if this association varied by ownership (i.e., for-profit, not-for-profit, government). The study population consisted of 9,513 freestanding NHs in 44 states in 2019. Average staffing levels were calculated for total nursing staff, as well as registered nurses, licensed practical nurses, and nurse aides. Regression results accounted for facility/resident characteristics (e.g., case-mix) and state-fixed effects. Across all ownership types, higher Medicaid rates were associated with higher staffing levels for all staffing outcomes, though average nursing staff levels are lower among for-profits and government owned NHs when compared to not-for-profits. Results also indicated that for-profit and not-for-profit NHs reacted to increases in Medicaid payment rates similarly (i.e., had higher staffing levels), whereas government NHs responded more strongly to payment increases. By raising Medicaid payment rates, policymakers can address staffing shortages by providing NHs with more financial resources, which in turn allows them to invest in additional nursing staff.

